# Effect of Edible Coating Enriched with Natural Antioxidant Extract and Bergamot Essential Oil on the Shelf Life of Strawberries

**DOI:** 10.3390/foods12030488

**Published:** 2023-01-20

**Authors:** Alessandra De Bruno, Antonio Gattuso, Davide Ritorto, Amalia Piscopo, Marco Poiana

**Affiliations:** 1 Department of AGRARIA, University Mediterranea of Reggio Calabria, 89124 Reggio Calabria, Italy; 2 Experimental Station for the Industry of the Essential Oils and Citrus Products SSEA, 89127 Reggio Calabria, Italy

**Keywords:** antioxidant, bergamot essential oil, citrus bergamia, coated fruits, pomace, edible coating, strawberry

## Abstract

In this study, the effects of the application of edible coatings on the shelf life of the strawberry were evaluated, with the aim of extending the fruit’s availability and shelf life while preserving its qualitative characteristics. In particular, the application of edible coatings enriched with a natural antioxidant to strawberries was evaluated for their physicochemical, microbial, and structural properties, during a storage period (up to 14 days) at refrigerated temperature. The experimental plan provided the formulation for edible coatings enriched with different concentrations of a natural antioxidant extract obtained from bergamot (Citrus bergamia Risso) pomace (1, 2.5, and 5%), bergamot essential oil (0.1% *v/v* and 0.2% *v/v*), and a synthetic antioxidant, butylated hydroxytoluene (BHT, 100 ppm). Moreover, a control test with untreated strawberries was considered. The enriched gum Arabic coatings provided good results related to the preservation of the qualitative parameters of the strawberries. The samples coated with the antioxidant extract (2.5%, sample D) and bergamot essential oil (0.1%, sample F) showed the best maintenance of the qualitative parameters after 14 days, showing lower decay rates (36% D and 27% F), good acceptability by consumers (between 5 and 6), and good retention of ascorbic acid (>30 mg 100 g^−1^).

## 1. Introduction

The consumption of fruits and vegetables has significantly increased in the last few years; indeed, diets rich in natural antioxidants are increasingly recommended for their beneficial effects on human health [1].

The problem with fruits and vegetables is their high perishability, which corresponds to a very short postharvest shelf life. They are very susceptible to postharvest quality losses due to mechanical damage, high respiration rates, microbiological damage, water loss, and natural physiological decay.

In relation to this, the correct management of the postharvest period and proper application of alternative practices leading to an extended shelf life are useful to improve the marketability of these natural and nutraceutical foods.

Strawberries (*Fragaria ananassa*) are among the most cultivated berries in the world. Recent data from 2022 reported 8,861,381 tons of production, a harvested area of 384,668 ha, and a yield of 230,364 hg/ha, showing increased cultivation in recent years all over the world [2]. Strawberries are commercially desirable worldwide due to their sweetness, flavor, and juiciness, and are largely consumed fresh or processed [3,4]. As reported by several authors [5,6], usually consumers prefer fruit with good appearance, flavor, durability, texture, and chemical characteristics. Strawberries are a good source of bioactive compounds such as organic acids, anthocyanins, phenols, flavonoids, sugars, vitamins, and minerals [7,8,9], which play an important role in human health. Strawberries are considered a “functional food”, offering different health benefits for human health, mainly attributed to their high content of phytochemicals and high antioxidant activity, acting directly on the modification of the etiology of chronic diseases [10]. In fact, their consumption helps in the prevention of cardiovascular and cardiometabolic diseases [11,12], mainly by improving insulin resistance. Afrin et al. [13] reported a large amount of bioactive compounds in strawberries, with important clinical aspects and great importance in human nutrition; as such, this fruit can be considered a functional food. Moreover, in view of the wide demand and consumption rates and for the qualitative and quantitative losses, in scientific research more and more attention is being paid the postharvest shelf life extension and to developing new alternative techniques to maintain quality, prevent losses and waste, and obtain lasting consumer acceptability in terms of the sensorial properties of the final product.

Strawberries, a being non-climatic fruit, have limited shelf-life ranges of 1–2 days at room temperature and 5–7 days at refrigerated temperatures [14,15,16]. Strawberries are highly sensitive to chemical and microbial deterioration; indeed, they may be contaminated during different phases such as harvesting, post- harvesting, or processing. The most common pathogens are hepatitis A virus [15] and enteric bacteria such as Salmonella and Escherichia coli [17]. Different technologies have been implemented with the aim of extending the shelf life of strawberries, such as refrigeration, modified atmosphere packaging, and alternative packaging, but in the last few years a lot of attention has been paid to edible coatings [16].

The application of a coating on the fruit’s surface generates a proximate zone with a modified atmosphere, which preserve the safety and nutritional quality because it delays ripening and protects the fruit from microbial disease and physiological senescence [18]. The performance of the edible coatings depends on their composition; they generally consist of biodegradable materials, such as plant extracts, proteins, lipids, and polysaccharides or blends of these materials [19]. Moreover, the edible coatings maintain the physicochemical (weight loss, pH, TSS, etc.) and antioxidant properties (phenols, vitamins, etc.) of the treated fruits and vegetables for longer periods [20].

Gum Arabic is a mixture of polysaccharides and glycoproteins (GPs) that is obtained from the Acacia Senegal tree and can be used as a glue and binder. For this reason, it is widely used in the food, beverage, pharmaceutical, and cosmetic industries as an emulsifier and a thickening agent [19,21].

Usually, synthetic or inorganic additives are used in coating applications, with good results, but modern consumers are more health-conscious and aware of the problems linked to toxicity for humans and the environment [22].

The actual trend is the application of natural coating materials and the addition of natural additives such as plant extracts as “green” additives to enhance the performance of the coatings [23]. These natural extracts can be obtained using different plant parts such as leaves [24] and fruit peels [25], or can be derived from by-products [26,27] and waste [28], and are rich in bioactive compounds with antioxidants properties [29].

Lotus leaf extract incorporated into edible coatings promoted the shelf life of goji berries more than a control sample treated with 1-methylcyclopropene (1-MCP) for about 4 days [30]. Other studies [19,31] including the use of natural extracts in coatings reported the extension of fruit shelf-life periods by combining the effect of the coating as a barrier against transpiration and the effects of the natural phenolic extract or essential oil incorporated into the polysaccharide matrix, with known antioxidant, antimicrobial, natural preservative effects [32,33,34]. Additionally, the application of essential oils (EO) is very important because they have a wide range of functional actions against foodborne and postharvest pathogens, as reported by Sanchez-Gonzalez et al. [35]. In particular, in this study bergamot essential oil (BEO) was used, which is obtained by rasping and cold-pressing the fruit peel. This essential oil was assigned the protected designation of origin “*Bergamot of Reggio Calabria*” (PDO, bergamot essential oil, 2015). BEO is characterized by an intense fragrance and freshness and is applied in several sectors, such as in perfumes, cosmetics, food, and confectionery. Moreover, BEO has shown antimicrobial, anti-inflammatory, analgesic, and antiproliferative properties [36,37].

The purpose of this research was to investigate the effects of edible coatings based on gum Arabic enriched with natural antioxidants on the qualitative properties of strawberries. In the context of the circular economy and sustainability, the new edible coating was formulated using natural antioxidants recovered from products and by-products derived from the bergamot production cycle, with the purpose of valorizing the citrus waste and extending the strawberries’ shelf life by improving the safety and quality during postharvest cold storage.

## 2. Materials and Methods

### 2.1. Materials and Chemicals

Strawberries (Cv. Camarosa) were collected in a local farm situated in Reggio Calabria (Italy) in April 2022, transported to the FoodTec laboratory of the University of Reggio Calabria, and immediately submitted to treatments. Firstly, the fruit were selected for uniformity in terms of their size, color, and weight, while the defective ones were discarded. Then, the fruit were dipped in a sodium hypochlorite solution (0.5%) for 2 min, washed with distilled water, and left to dry for 1 h near a UV area in a laminar vertical flow hood (UV lamp 30 W, mod. ASALAIR 1200 FLV, Asal Srl, Milano, Italy) at room temperature under forced air (20 °C).

**Figure 1:**
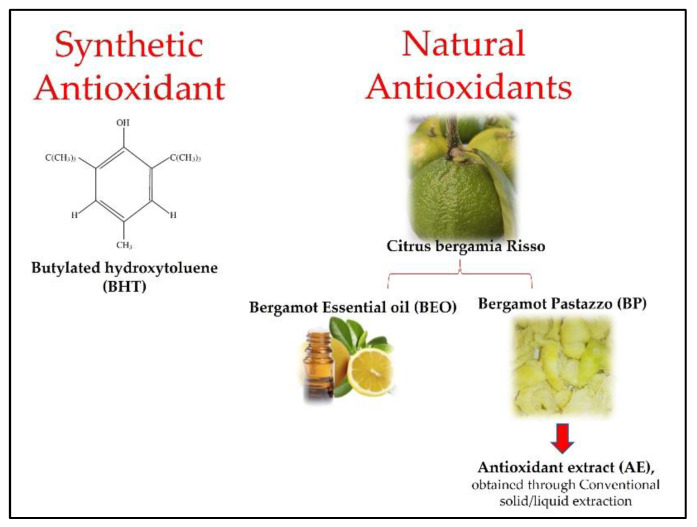
Synthetic and natural antioxidants used to enrich the edible coatings.

Both bergamot “pomace” (BP) and bergamot essential oil from Reggio Calabria DPI (BEO) were sourced by a company located in Reggio Calabria (Italy). BP is a by-product of citrus fruit processing (Citrus bergamia Risso) during juice production, comprising the peel, pulp, and seeds (Figure 1). The BP was dehydrated at 50 °C in a tangential air-flow cabinet (“Scirocco” model, Società Italiana Essiccatoi, Milan, Italy) until reaching a 12% moisture content and was then powdered. The BEO was obtained by rasping and cold-pressing the fruit peel.

The butylhydroxytoluene (BHT) was purchased from Merck KGaA (Darmstadt, Germania).

### 2.2. Preparation and Characterization of Antioxidant Compounds from Bergamot Pomace (AE) and Bergamot Essential Oil (BEO)

The antioxidant extract (AE) was obtained following the method reported by Imeneo et al. [27] and appropriately modified. Briefly, 100 g of BP was mixed with 400 mL of ethanol/water (1:1, *v*:*v*) solution and kept under continuous stirring (30 min, 70 °C) using a conventional solid–liquid extraction method. Subsequently, the AE was centrifuged (8000 rpm, 10 min, 4 °C) in a refrigerated centrifuge (NF 1200R, Nüve, Ankara, Turkey), filtered through 0.45 µm filter paper, and stored at 4 °C until use. The AE was characterized for its total phenolic and flavonoid contents, and the antioxidant activity was measured following the method reported by Imeneo et al. [27].

The bergamot essential oil (BEO) characterization process was carried out according to the modified methodology reported by Gionfriddo et al. [38]. Briefly, the composition was determined through a GC2010 gas chromatograph equipped with a flame ionization detector (FID) and a capillary column of fused silica (DB-5MS). The temperature program was 70 °C for 10 min, then heating at 3 °C/min to 120 °C, heating from 130 °C to 220 °C at 4 °C/min, maintenance for 5 min at 220 °C, heating from 220 °C to 280 °C at 15 °C/min, and maintenance for 10 min at 280 °C. The operative conditions were as follows: split ratio of 1:60 at 230 °C, helium as the carrier gas, with a flow rate of 1.5 mL/min; FID 250 °C; injection volume of 0.2 µL, manually injected in split mode. The main constituents were identified by comparing their RI (retention index) values with those provided in the literature and the internal standards. 

### 2.3. Coating Preparation and Application on Surfaces of Strawberries

**Figure 2:**
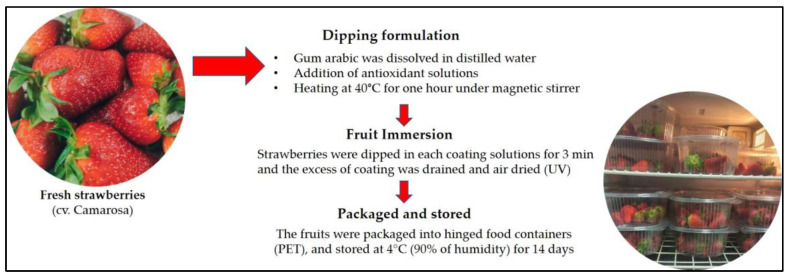
Schematic overview of the sample preparation process.

As reported in Figure 2, the experimental plan involved the formulation of six different tests, in addition to the control conditions (untreated sample: CTRL).

The coating was prepared as follows [39]. The gum Arabic (2% concentration, *w/v*) was dissolved in distilled water until it was completely dissolved, after which the AE, BEO, and BHT solutions were added and heated at 40 °C for one hour with a magnetic stirrer. Subsequently, 1% glycerol (*v/w*) was added as s plasticizer to improve the strength and flexibility of the coating solutions. Tween 20 (0.5% *v/v* OE tween 20/OE) was added to promote the dispersion of the essential oil. The concentrations of AE, BEO, and BHT added to the coatings are reported in Table 1 and here: 100 ppm of BHT (sample B); 1% AE (sample C); 2.5% AE (sample D); 5% AE (sample E); 0.1% BEO (sample F); 0.2% BEO (sample G).

The strawberries were dipped in each coating solution for 3 min and the excess of the coating was drained and air-dried (under UV and at room temperature to prevent environmental contamination). The fruit samples were packaged into hinged food containers (PET) and stored at 4 °C. Each treatment contained three replicates.

Before the application of the coating on the strawberries, the different solutions were analyzed for their antioxidant components to define the real transfer or enrichment of the bioactive compounds.

### 2.4. Effect of Edible Coating Application based on the Physicochemical Analysis

All coated strawberries were subjected to a physicochemical analysis at different times (1, 3, 7, 10, and 14 days) during the storage period.

#### 2.4.1. Weight Loss Percentage

The strawberries were individually weighed (11 reruns for each sample) at the storage times. The weight loss was calculated as the difference between the initial and final weights of the fruit and the values were reported on a percentage basis in accordance with the AOAC standard method [40].

#### 2.4.2. Decay Percentage

The decay percentage (DC) was evaluated at each storage time following the method reported by D’Acquino et al. [41]. The strawberries were visually evaluated and considered decayed when visible damage, described as brown spots, softening, or mold growth, was detected. The DC was calculated using the following equation (Equation (1)):

DC = (NIF/INF) × 100(1)

where NIF is the number of infected fruit samples and INF is the initial number of all fruit samples.

#### 2.4.3. Surface Color Measurement

The surface color was measured at ten points for each sample using a Minolta CM-700d Spectrophotometer, with reference to CIE L*a*b* coordinates using a D65 illuminant [42]. These values were then used to calculate the hue degree (h°) and chroma (C*), as reported by Hernández-Muñoz et al. [43].

#### 2.4.4. Texture Analyses: Penetration Test

The strawberry fruit texture was determined using a TA-XT Plus Texture Analyzer (Stable Micro Systems Ltd., Godalming, UK), following the method reported by Doving et al. [44] with appropriate modifications. Data acquisition and curve integration were carried out using Exponent software 6.1.4.0 (Stable Micro Systems Ltd., Godalming, UK).

The penetration test outlined a mechanical force displacement using a 5 kg loading cell and with a cylindrical flat head probe with a diameter of 5 mm (P/5) entering the fruit (placed on the plate with the receptacle cavity upright to the compression probe to assess its firmness). The mechanical profiles were acquired with a data acquisition rate of 300 pps with the following instrumental settings: pretest speed: 10.00 mm/s; test speed: 5 mm/s; post-test speed: 10.00 mm/s; trigger force: 2.0 g. For each sample, ten replicates were used.

#### 2.4.5. Sensorial Analysis

The sensorial analysis test was based on perceptions of visual appearance (hue, brightness, integrity), aroma intensity (fruity, aromatic intensity, citrus, fermented), taste (sweetness, acidity, bitterness, aftertaste), and texture (turgidity, juiciness) and the overall acceptability of the fruits. It was carried out on strawberry samples at each monitoring time (0, 7, and 14 days). Eighteen panelists (between 25 and 64 years old) with previously experience in sensory analyses were trained before the sessions to identify the gustatory attributes to be evaluated. Each panelist was also asked to evaluate the general acceptability from a consumer point of view. The sensorial analysis was based on a 0-to-9-point hedonic scale. A score of 4.5 was considered the limit of acceptability.

#### 2.4.6. Determination of Total Soluble Solids (TSS), pH, Titratable Acidity (TA), Organic Acids, and Microbiological Counts

For the determination of the chemical parameters, about 100 g of fruit was randomly taken from each sample and homogenized using an Ultra-Turrax (T 25 digital, IKA, Staufen, Germany), with the aim of obtaining a homogenate sample. The TSS was determined using a digital handheld refractometer (DBR 047 SALT), and the results were expressed in degrees Brix (°Bx). The pH value of the strawberry samples were measured at 25 °C using a digital calibrated pH meter (pH 4, pH 7; Crison Basic 20, Spain) according to the AOAC [44,45,46].

For the TA determination, 5 g of homogenate was diluted with 100 mL of deionized water and then titrated with 0.1M NaOH. The end-point reading was monitored using a pH meter (Crison Basic 20, Spain). The results are expressed as citric acid % values [47].

The organic acid extraction process was performed according to Ikegaya et al. [48], with some modifications. An aliquot of 5 g of strawberry homogenate and 25 mL of distilled water was blended using an Ultra-Turrax. Then, the mixture was mixed for 30 s with a vortex and centrifuged at 9000 rpm and 4 °C for 10 min in a refrigerated centrifuge (NF 1200R, Nüve, Ankara, Turkey), then the supernatant filtered with a PTFE 0.45 µm (diameter 15 mm) syringe filter. The concentration of organic acids was determined followed the method reported by Panebianco et al. [49]. The analysis was conducted using a Knauer HPLC Smartline Pump 1000, equipped with a Knauer Smartline UV Detector 2600 set at 210 nm, using an Acclaim OA5 column (4 mm i.d. × 250 mm length × 5 µm particle size). The chromatographic analysis was carried out in isocratic conditions using as the mobile phase 100 mM of Na_2_SO_4_ acidified to 2.65 pH with methane sulfonic acid CH_3_SO_3_H (30 °C; flow rate: 0.6 mL/min). For the quantification of each organic acid, external standards were used, and the results are expressed as mg 100 g^−1^ of fresh strawberries (mg 100 g^−1^).

For the microbial analysis, each sample was serially diluted (1:10) in a Ringer solution and homogenized using a stomacher (BagMixer^®^ 400 P, Interscience, France) for 2 min; subsequently, 1 mL of each dilution was transferred onto the surfaces of the plates used.

Dichloran Rose Bengal Chloramphenicol (DRBC) agar base plates were used for the enumeration of yeasts and molds, and the plates, after solidification, were incubated at 25 °C for 4–5 days before counting the colonies. The total bacteria count (TBC) was performed by inoculating ready-to-use chromogenic plates (Compact Dry) and incubating them at 25 ± 2 °C for 48 ± 3 h. The results are reported as Log10 colony-forming units (CFUs) g^−1^ of strawberries.

### 2.5. Antioxidant Properties of Strawberries

#### 2.5.1. Extraction of Antioxidants Compounds

The extraction of antioxidant compounds was carried out according to Mustafa et al.’s [50] method, with some modifications. First, 5 g of strawberry homogenate was mixed with 10 mL of an acidified (pH 3, HCl) mixture of EtOH/H_2_O (70:30 *v/v*). After, the mixture was mixed for 30 s in a vortex and placed in an ultrasonic bath for one hour (40 kHz, 25 °C, 50% power). The strawberry extracts (SE) were centrifuged at 9000 rpm for 10 min at 4 °C, then the supernatants were filtered with a syringe filter (RC, 0.45 µm, diameter 15 mm) and used for the analysis.

#### 2.5.2. Total Phenolic Contents (TPC)

The TPC were determined following the method reported by Letaief et al. [51], with appropriate modifications. In a volumetric flask measuring 25 mL, 0.1 mL of AE or SE (for each sample), 9 mL of deionized water, and 0.5 mL Folin–Ciocalteau reagent were shaken vigorously and kept at room temperature. After 5 min, 5 mL of Na_2_CO_3_ solution (5% *w/v*) was added, brought up to volume with water, and incubated for 60 min. The absorbance was measured at 765 nm versus a blank (sample replaced by water). The total phenolic content was expressed as mg gallic acid equivalent L^−1^ of AE (mg GAE L^−1^) and mg gallic acid equivalent 100 g^−1^ of fresh strawberry (mg GAE 100 g^−1^ F.W.).

#### 2.5.3. Total Flavonoid Content (TF)

The total flavonoid content (TF) was determined following the method reported by Papoutsis et al. [52], with slight modifications. In brief, in a volumetric flask measuring 5 mL, 300 µL of AE or SE (for each sample), 1000 µL of distilled water, and 150 µL of NaNO_2_ (5%, *w/v*) were mixed and kept in dark conditions for 6 min. Then, 150 µL of AlCl_3_ (10%, *w/v*) was added and incubated for 6 min at room temperature. Subsequently, 2000 µL of NaOH (1 M) was added, then at the end the water was used to complete the volume. The same solution without the sample was used as a blank and the absorbance was measured at 510 nm. The results are expressed as mg of catechin equivalents L^−1^ of AE (mg CE L^−1^) and mg of catechin equivalents 100 g^−1^ of fresh strawberry (mg CE 100 g^−1^ F.W.).

#### 2.5.4. Total Antioxidant Activity (TAA): DPPH and ABTS Assays

The antioxidant assays (DPPH and ABTS) were performed using the method reported by Imeneo et al. [27], which was appropriately modified.

##### DPPH Assay

Here, 10 µL of AE or SE (for each sample) was allowed to react with 2990 µL of 6 × 10^−5^ M of methanol solution of DPPH under darkness at room temperature for 15 min. The absorbance was measured at 515 nm against methanol as a blank, using a double-beam ultraviolet-visible spectrophotometer (Perkin-Elmer UV-Vis _2, Waltham, MA, USA).

##### ABTS Assay

Here, 10 µL of AE or SE (for each sample) was added to 2990 µL of the ethanol solution of ABTS^+^. The absorbance was measured after 6 min in the dark using a spectrophotometer. The blank was prepared with EtOH.

For both the antioxidant assays (DPPH and ABTS), the quenching of the initial absorbance was plotted against the Trolox concentration (from 3 to 18 µM) and the results were expressed as mmol Trolox L^−1^ of AE (mmol TE L^−1^) and mmol Trolox kg^−1^ of fresh strawberries (mmol TE kg^−1^ F.W.).

#### 2.5.5. Total Anthocyanins Content (TAC)

The TAC extraction was performed following a different pH colorimetric method. Here, 2 g of strawberries and 10 mL of HCl-acidified methanol (99.9/0.1 *v/v*) were left in the dark overnight under refrigerated conditions (4 °C). Subsequently, the extract (TCAE) was centrifuged at 6000 rpm for 5 min and filtered through 0.45 µm filters.

The TAC analysis was performed according to Tahir et al.’s [53] method, with some modifications. Briefly, 0.5 mL of the TCAE (for each sample) was reacted with 4.5 mL of the two different pH buffer solutions—the first one with potassium chloride buffer (0.025 M, pH = 1.0) and the second with sodium acetate buffer (0.4 M, pH = 4.5)—then kept at room temperature for 15 min. The absorbance was recorded at wavelengths of 510 and 700 nm against a blank (HCl-acidified methanol (99.9/0.1 *v/v*) using a double-beam ultraviolet-visible spectrophotometer (Perkin-Elmer UV-Vis λ2, Waltham, MA, USA). The quantification of total anthocyanins was calculated using Equation (2) and determined as mg 100 g^−1^ of fresh weight (F.W.) of pelargonidin-3-glucoside (PGN), the anthocyanin predominant in strawberries, as reported by Sarıdaş et al. [54]:

TAC (mg/100 g) *=* (A × MW × DF × V × 100)/(ε × d × m) (2)

where:

MW = molecular weight of pelargonidin-3-glucoside (433.2 g/mol);
DF = dilution factor;
V = extract’s volume;
ε = coefficient of molar absorptivity of pelargonidin-3-glucoside (31,600 L/cm/mol);
d = path length (1 cm);
m = mass of the sample (g).

#### 2.5.6. Identification and Quantification of Individual Antioxidant Compounds

For the chromatographic analysis of individual antioxidant compounds, the method reported by Romeo et al. [28] was followed. Here, 5 µL of AE (for each sample) was injected in a UHPLC PLATINblue instrument (Knauer, Berlin, Germany) provided with a binary pump system, using a Knauer blue orchid C18 column (1.8 mm, 100 × 2 mm) coupled with a PLATINblue PDA–1 (photo diode array detector) (Knauer, Berlin, Germany) and Clarity 6.2 software. A gradient elution program was used (0–3 min, 5% B; 3–15 min, 5–40% B; 15–15.5 min, 40–100% B), where the mobile phases were (A) water acidified with acetic acid (pH 3.10) and (B) acetonitrile. For the quantification of each antioxidant compound (p-cumaric acid, ferulic acid, eriocitrin, neoeriocitrin, narirutin, naringin, neohesperidin, melitidin, and brutieridin), external standards were used, and the results are expressed as mg L^−1^ of AE (mg L^−1^). 

### 2.6. Statistical Analysis

The results obtained in this experiment are shown in the tables and figures as means ± SDs of different measurements. The statistical differences were evaluated using a one-way analysis of variance (ANOVA) with Tukey’s post hoc test (*p* < 0.05), performed using SPSS software (Version 20.0, SPSS Inc., Chicago, IL, USA).

## 3. Results and Discussions

### 3.1. Characterization of Antioxidant Extract (AE) and Bergamot Essential Oil (BEO)

In this work, several edible coatings were formulated with different concentrations of antioxidant extracts obtained from bergamot pomace (AE) and bergamot essential oil (BEO), as each enrichment compound might influence the characteristics and properties of the film or of the packaged food, as reported by Ganiari et al. [55]. For this reason, it is very important to confirm that both AE and BEO show antioxidant activity.

The first step of the experimentation involved the extraction of the antioxidant compounds from bergamot pomace and their subsequent physicochemical characterization. The main results are reported in Table 2.

The AE showed pH values of about 3.18 and 21.1 °Brix. The low pH value can be considered a good result, as it allows the natural acidification of the substrate on which it will be applied. Regarding the evaluation of the antioxidant activity, spectrophotometric and chromatographic methods were applied, which highlighted the high TPC (7751 mg GAE L^−1^) and TFC (2783 mg CE L^−1^) values, while a good total antioxidant activity level was revealed through the ABTS assay (2100 mmol TE L^−1^).

The main individual phenolic compounds identified in the AE, and in order of their determined concentrations, were neoeriocitrin, naringin, neohesperidin, brutieridin, melitidin, p-cumaric acid, eriocitrin, ferulic acid, and narirutin.

Regarding the BEO, the total antioxidant properties and the volatile fraction were evaluated, and the data are reported in Table 3.

Two total antioxidant assays were tested to evaluate the properties of the BEO. The obtained results highlighted that the BEO was able to decrease the DPPH and ABTS free radicals, showing similar results between the two assays (about 230 and 267 mmol TE L^−1^, respectively).

An evaluation of the volatile fraction is very important to define the quality of an essential oil. The two main compounds present in the BEO were limonene (46.727%) and linalyl acetate (34.151%), which showed values similar to those reported by Gionfriddo et al. [35]. The composition of the BEO may vary according to different parameters, such as the harvest period and geographic origin [56].

### 3.2. Effect of Edible Coating Application on Physicochemical Properties of Minimally Treated Strawberries

#### 3.2.1. Weight Loss

Weight loss represents an important aspect that can be used to evaluate the quality of fruit, and it is related to the transpiration and respiration of the fruit [16]. Strawberries are easily susceptible to water loss, which causes contraction and weakening of the fruit tissue due to their very thin skin. This has negative effects on the appearance of the fruit, causing changes in texture (softening), color, and aroma and accelerating senescence, pathogen development, shriveling, and chilling injury [57], consequently causing economic losses. The edible coatings provide a barrier function by protecting the fruit from the external atmosphere, but also by limiting transpiration by delaying dehydration, providing a qualitative improvement in weight loss.

In this study, the weight loss % increased gradually in all samples during storage (Table 4), showing significant differences (*p* < 0.01) both among the treatments and among the monitoring times.

The strawberries without coating (samples A), among all the treated samples, showed the largest decay percentage from day 3 and significantly differed at day 14 (25.38%). Meanwhile, samples D and F responded better to the coating, showing decay values of about 12% at the end of the monitoring period.

#### 3.2.2. Decay Index

**Figure 3:**
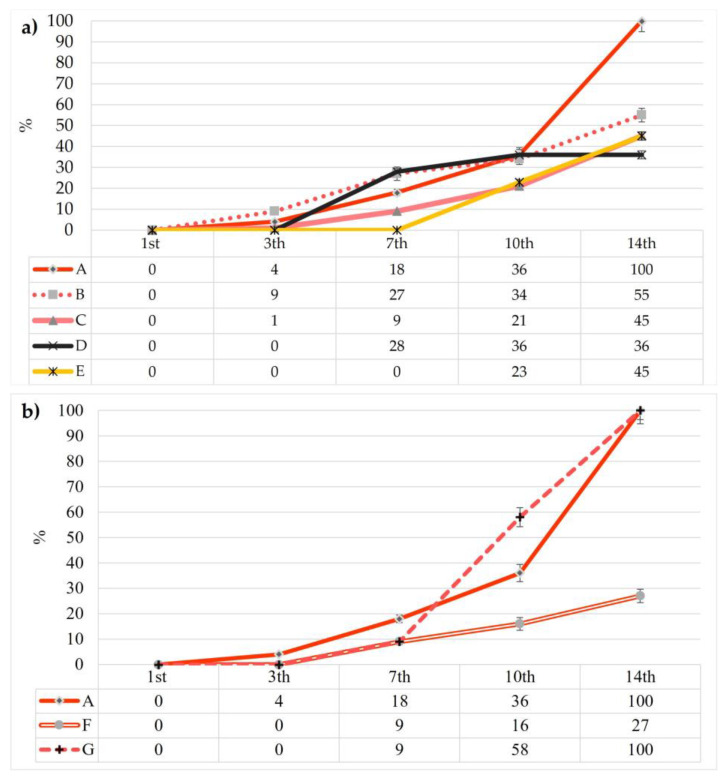
Effects of edible coatings enriched with AE (**a**) and BEO (**b**) on decay index values of strawberries during storage.

The decay index of coated strawberries was evaluated during the storage period, and the results are reported in Figure 3. The decay index increased during the storage period for all treatments; only the E sample at seven days of storage showed a decay percentage of 0%. At the final monitoring time, samples F (27%) and D (36%) showed lower decay index values, followed by C and E with a DP of 45% and B with 55%. Total decay was shown by sample A, namely the control. The application of the edible coating caused a decreased decomposition rate during storage, as also reported by Agapito-Ocampo et al. [16]. Even sample G showed total decay at 14 days of storage, probably due to the negative effect of the higher concentration of BEO added to the coating formulation. These results were in accordance with the expectations of the dipping solution enrichment with AE and BEO to perform the antioxidant action, coupled with the barrier effect of the coating. Therefore, for this parameter, the best result was obtained by sample D, which was the sample treated with an edible coating enriched with AE at a medium concentration (Figure 3a), followed by sample F, the sample coated with gum Arabic and 0.1% BEO.

#### 3.2.3. Effects of Edible Coatings on the Texture of the Strawberries

**Figure 4:**
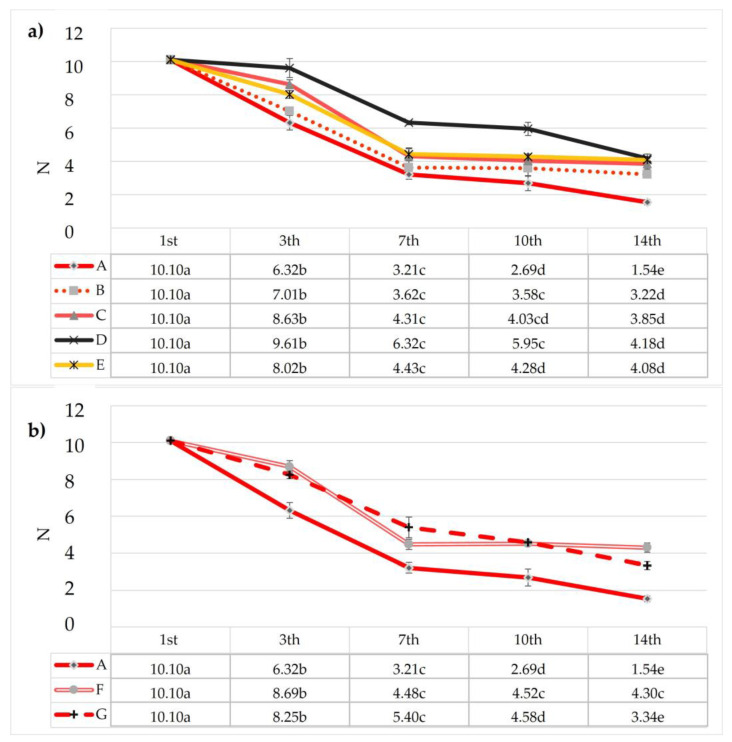
Effects of edible coatings enriched with AE (**a**) and BEO (**b**) on the firmness of strawberries during storage. Small letters within a column show a significant difference as assessed by Tukey’s post hoc test.

The fruit firmness was analyzed on the coated samples during the storage time (Figure 4a,b), because it represents one of the essential parameters to determine fruit quality. The softening is a natural physiological effect of fruit ripening with cell wall changes and the dissolution of the middle lamella, which in turn causes loss of cell-to-cell adhesion [58,59,60]. Strawberries are more perishable and subjected to mechanical damage, pathogen attacks, and quality losses during storage.

At time 0, the firmness of the fruit was 10.1 N, while after seven storage days, decreased firmness was observed, both in the uncoated and coated samples, but the lowest value was found in the control sample (3.21 N), similar to sample B (3.62 N). The highest firmness values were recorded in samples D and G at 6.32 N and 5.4 N, respectively. Additionally, after 14 days of cold storage, samples A and B showed the lowest firmness values as compared with the other samples. The firmness results for samples A and B were 1.54 N and 3.22 N, respectively, which were very low values in terms of acceptability. The firmness is influenced by the softening of the fruit. The results were in accordance with the study by Tahir et al. [19], where the retention of flesh firmness of blueberries was achieved by the combined effect of African baobab pulp extract and GA, while Kahramanoğlu et al. [61] reported that during storage there was a greater decrease in firmness in untreated strawberries than in those treated with an extract incorporated in the coating. The standard deviations were high in many samples, but this is normal due to the hardness variability under the same conditions, in agreement with Doving et al. [44]. For this reason, we used ten replicates for each treatment.

#### 3.2.4. Surface Color Measurement

In Table 5, the results related to the surface color are reported. The statistical analysis (ANOVA) did not show significant differences (*p* > 0.05) during the storage period (until 14 days) for the L*, a*, b*, or chroma (C*) in any of the coated samples.

Only the control (sample A), or the untreated sample, showed a decreasing trend during the storage period (*p* < 0.05), which was already apparent from the 7th day. Instead, the hue angle (H*) of the strawberries, which indicates the maintenance of the fruit’s natural color characteristics, decreased during the storage period meaningfully in some samples (A, B, E, F), particularly in the control sample (A).

The L* parameter is an indicator of fruit lightness, and the results showed that the coated samples had reduced values compared to the control. In fact, the L* value of the strawberry surfaces was highest for the untreated sample (49.89), although all dipped samples showed similar values. This reduction may have been due to the presence of gum Arabic; in fact, Tahir et al. [39] also reported that their coating with the lowest amount of GA (10%) had the highest L* value as compared to another coating with a higher concentration (15%).

The highest value of the a* parameter was found in the control sample, and in the same sample there was a big loss of red tone during the storage time, probably due to the faster perishability of the strawberries. In the sample with the lowest BE content (sample C), the value of a* decreased linearly from 15.78 at T0 to 14.99 at T7 and 14.15 at T14. This showed that the coating treatments, as compared to the control fruit, maintained their red color over time. This may have been associated with the retarded biosynthesis in the metabolic activity of the red pigment during ripening, particularly pelargonidin-3-glucoside, which is responsible for the red color.

Regarding parameters b* and C*, the treated samples maintained stable parameters, with both displaying non-consistent and insignificant variations during the overall prolonged storage. A change in color is considered an indicator of ripening in fruit [62]. It is linked to physiological processes. Considering the reductions in redness, yellowness, and chroma in the untreated samples during storage, the data suggested that the coating preserved the ripening factors correlated with changes in color. However, postharvest color changes do not affect the shelf life of strawberries, as reported by Ktenioudaki et al. [63].

#### 3.2.5. Sensorial Analysis

The sensorial characteristics of minimally treated strawberries were determined by a group of regular consumers of this fruit. The main aspects revealed by the panel group are shown in Table 6, and the results are reported as the median values of all scores. The average score of 4.5 was considered the limit of acceptability for the fruit, as also reported by Garcia et al. [64], and in the table we report only the initial (1st day) and final (14th day) results evaluated in the samples.

At the first timepoint, the overall acceptability of the samples was good, with an average higher than 6 in all samples, including the control (A). It was possible to highlight that the application of the edible coating caused a variation in aromatic intensity compared with sample A. The film that forms on the surface of the fruit probably limits the release of aromatic components. At the end of the storage period (14 days), the coated fruit samples were subjected to significant variation, which determined the end of their shelf life. At 14 days, only some samples fell within the limit of 4.5, including samples D, E, and F, namely those formulated with the addition of AE and BEO.

The addition of higher concentrations of BEO to the coating formulation causes greater persistence of the aroma, causing an alteration of the organoleptic properties of the final product.

#### 3.2.6. Total Soluble Solid (TSS), pH, Titratable Acidity (TA), and Organic Acid Levels of Coated Strawberries

**Figure 5:**
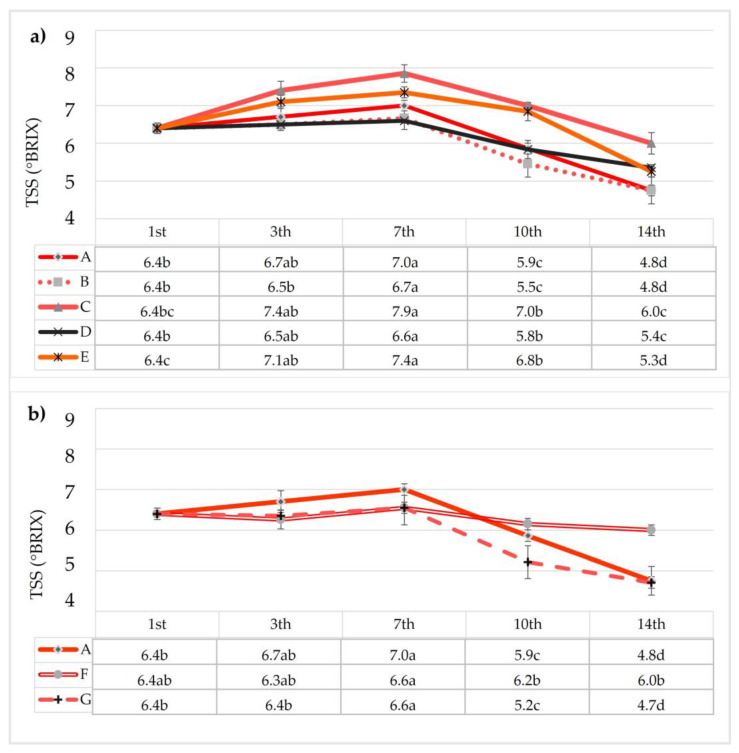
Effects of edible coatings enriched with AE (**a**) and BEO (**b**) on total soluble solids (TSS) of strawberries during storage. Small letters within a column show a significant difference as assessed by Tukey’s post hoc test.

The results related to the total soluble solids (TSS) during storage are reported in Figure 5. At the beginning of the experiment, the strawberries showed a TSS content equal to 6.4 °Brix; during storage, the values tended to increase in all treated samples from the 3rd day onwards, reaching a peak at 7 days in all samples. From the tenth day of storage, onwards, reductions in TSS content were observed. The changes in TSS content during this time were confirmed by the ANOVA test, whereby all samples showed significant differences, although only sample F showed lower statistical differences (*p* < 0.05). The initial increase and subsequent reduction in TSS were due to the hydrolysis of carbohydrates during fruit maturity [65] and from their consumption for respiration. Sugars are accumulated during ripening and then decline during senescence.

Moreover, in accordance with Bahmani et al. [66], the delay in the TSS could be considered an indicator of over-ripening and senescence. Among the samples, samples A and B showed faster decay.

As shown in Table 7, the pH values of the strawberries varied statistically during the storage period (*p* > 0.05), with the values ranging between 3.4 (1st day) and 4 (14th day). The obtained pH values were in accordance with Agapito-Ocampo et al. [16]. The pH values increased during the conservation time evenly in all test samples. Samples D and E were the samples that changed less after 14 days.

The highest pH levels were observed in samples A and B at 14 days (3.97 and 4.04, respectively), as compared with the samples treated with coatings enriched with AE and BEO. These results may have been due to the effects of enzymatic activities and the ripening of the strawberries. These results are in agreement with those found by Gol et al. [67], who detected a greater increase in pH in uncoated strawberries than in coated samples. Moreover, this tendency could be due to the consumption of organic acids during fruit ripening.

As is clearly visible in Table 7, the titratable acidity (TA) shows a different trend in the treated samples compared to the control (A). Indeed, in sample A, the TA increased at 7 days of storage and then decreased significantly at 14 days; on the contrary, in the coated strawberries, the TA values tended to statistically increase during the conservation period in samples C, F, and G.

With reference to fruit ripening, the TA values did not show changes related to the consumption of organic acids in the coated samples. This effect was more visible in sample A, in which high significant decay at the end of shelf life was shown. This effect was found in the literature according to Dìaz-Mula et al. [68], who explained the higher acidity loss in uncoated fruits as being due to their high respiration rate during storage, which affects the organic acids’ respiratory activity (Krebs cycle). The synthesis of organic acids happens during fruit maturation [69], consequently causing increased acidity and decreased senescence. The obtained results highlighted how the edible coating preserved and improved the TA values; therefore, good flavor was found because of the high TA and TSS values. The data showed a low TA value in the control sample at the end of study (0.48 mg 100 g^−1^ CA), confirming the loss of quality and advance to senescence. The TA results were in accordance with Jouki and Khazaei [70], who observed similar average values.

Ascorbic acid (AA), or vitamin C, is one of the major components of strawberries, and its content is an indicator of quality relevant to define freshness of fruits [71]. Many authors have reported that decreased AA during storage is caused by its oxidation [72] and the respiration rate of the fruit [73]. The use of a coating promotes protection against both effects. As is possible to see in Table 7, the results obtained in this study promote this effect. The control sample (A) showed significant variation of the AA content values during the storage period (*p* < 0.01), with the lowest value being shown at 14 days. The initial AA content was 33.01 mg 100 g^−1^, and after seven days it decreased to 28.35 mg 100 g^−1^, while at 14 days it was 27.02 mg 100 g^−1^ (the lowest detected value). These results are confirmed in the literature, where Khodaei et al. [74] reported a similar trend with delayed vitamin C deterioration over time compared with a control sample. The highest values compared with T0 were recorded in sample G, which might have been due to continued ripening. At 14 days, the AA contents decreased in all samples, particularly the control sample (A), the sample treated with BHT (B), and the samples dipped in the solutions with the highest amounts of BE and BEO (G), with values of 27.02, 29.07, 29.82, and 29.94 mg 100 g^−1^, respectively. Samples C, D, and F had the highest amounts of AA.

Citric acid is the predominant organic acid in strawberries. Some authors have reported that citrate’s synthesis is linked to fruit respiration during the different stages of physiological growth, followed by a reduction during strawberry ripening [75]. The data detected during this experimentation process are reported in Table 7, where one can observe that there were great losses (highly significant, *p* < 0.01) of this organic acid, particularly in the control sample (A), from 692.5 mg 100 g^−1^ at the beginning to 402.9 mg 100 g^−1^ at the end of the shelf life (14 days).

Regarding the trend shown during the storage period, the CA contents varied significantly only in three samples, A, B, and G (*p* < 0.01); all other samples showed no significant differences (*p* > 0.05). All coatings enriched with AE highlighted the good stability of this acid during the period.

#### 3.2.7. Microbiological and Sensorial Parameters of Minimally Treated Strawberries

Generally, minimally treated fresh fruit have a short shelf-life range (4–7 days), which is very important to preserve the freshness of the fruit and avoid excessive losses due to the reduction in their quality; for this reason, the evaluation the microbiological and sensorial parameters is very important.

As the minimally treated fruit are not subjected to thermal treatment, they should be processed and stored at temperatures below 5 °C, with the aim of extending their shelf life and microbiological security. Their composition makes them a favorable substrate for the growth and development of some microbial forms, such as molds and yeasts [76].

In Table 8, the microbiological results are reported. The CBT, yeasts, and molds were revealed already from the 1st monitoring day in samples A (control) and B, while the other samples did not show any contamination. The use of plant extracts and essential oil in edible coatings should provide advantages to preserve their high sensibility to microbial decay. The obtained microbiological values fall within the acceptable limits set by the French regulations, which include a maximum aerobic plate count of 5 × 107 cfu/g at the end of shelf life for different fresh-cut vegetables [77].

During the conservation period, increases in all analyzed microbiological parameters were observed, particularly at 14 days of storage. The samples more subjected to microbiological deterioration were samples A, B, and C; these values showed that the application of edible coatings is useful to improve and extend the quality of strawberries.

#### 3.2.8. Antioxidant Activity of Minimally Treated Strawberries

Strawberries contain approximately 390 mg of total phenols per serving and are classified in 9th place on the list of the 100 richest sources of dietary phenols, with high antioxidant activity, as reported by Mustafa et al. [50]. The more studied group of phenolic compounds in strawberries is that of anthocyanins, which are responsible for the red color of the fruit. The other classes of phenolics that characterize the strawberries are the tannins, flavonols, and esters of hydroxycinnamic acids [50]. Therefore, strawberries are rich in phenols and antioxidant compounds, making them a good health indicator, and the relative results obtained for their evaluation are reported in Table 9.

The TPC values increased during postharvest storage as a direct response to fruit ripening and depending on several factors that may influence their biosynthesis and availability [66,78,79]. Significant differences were noted during the storage period but no variation was noted among the coated samples.

The monitored data from the refrigerated storage period exhibited similar increments of TPC values after 7 days in all samples, which in almost all cases were maintained up to 14 days. Only samples D, E, and G decreased slowly (*p* < 0.05) after seven days. The reduction in TPC values during this period may have been due to the possible breakdown of the cellular assembly and structure as a consequence of fruit senescence [80]. Similar values were reported for sample G, which was treated with 0.2% of BEO, in accordance with Shirzad et al. [81], who reported a similar effect of essential oil on the cell walls of fruit with ageing and an increase in the enzyme activity of the polyphenol oxidase enzyme.

Moreover, the TPC values for all other samples were higher than the control, which may have been due to the protective barrier formed by the coatings on strawberries’ surfaces reducing the enzymatic effect on the oxidation of phytochemicals, with a consequent loss of quality [82,83].

The effects of the edible coating treatments on the total flavonoid content (TF) of the strawberries are illustrated in Table 9. In all samples, the TF increased over time, except for the sample coated with the lowest quantity of BEO dipping solution (F), in which no statistical differences were found over time. Significant differences were revealed among the different coated samples, with high values found in sample B at the 14th day (35.8 mg CE 100 g^−1^). Similar values were recorded in the samples with the highest amounts of AE and BEO (samples E and G), with 27.59 and 26.19 mg CE 100 g^−1^, respectively. The obtained results are in accordance with Chen et al. [84].

The results related to the anthocyanin concentrations determined on the tested samples are presented in Table 9. During the storage period, the samples showed a different trend, with significant differences (*p* < 0.01), while only samples C and G preserved the same contents of anthocyanin during the monitoring period (14 days).

The control sample (A) showed the lowest TAC concentration of 20.34 mg 100 g^−1^, in agreement with Zheng et al. [85] and Tahir et al. [19], as compared with the coated samples. All tested samples showed an increase in the TAC at 7 days of storage, particularly sample A (33.74 mg 100 g^−1^), but the same sample underwent a faster decrease during the following storage days (17.52 mg 100 g^−1^). The obtained data highlight that the application of an edible coating on the strawberries preserves the TAC. For sample G, even though it presented the lowest TAC, it maintained constant levels of anthocyanins during the time period, with no statistical differences, and with the lowest TAC after sample A. This was probably due possible to an excess of BEO, which has negative effects on metabolic activity and on cell membranes [86], promoting senescence. Samples B and C at the end of the shelf life displayed similar values compared to the beginning, while samples D, E, and F showed the highest TAC values (23.94; 24.96; 24.85 mg 100 g^−1^). At 14 days of storage, the samples showed a reduction in TAC, which was probably associated with fruit senescence, leading to the inhibition of anthocyanin biosynthesis, as also reported by Wang et al. [87] and Khodaei et al. [74].

Different values were highlighted between the two antioxidant tests, each of which evaluated different reaction mechanisms; in fact, the results were different, with a higher total antioxidant activity (TAA) revealed by the ABTS assay (Table 9).

The DPPH radical scavenging capacity assay showed a different trend among the samples, with the highest values being obtained after 7 days, in particular in samples E and F, which showed values of 205.06 and 215.53 mmol TE kg^−1^ in strawberries, respectively, which slightly decreased during storage. Indeed, at 14 days, the assay showed similar values to the 1st day. No significant differences (*p* > 0.05) were evidenced for samples A, D, and G, for which the total antioxidant activity levels determined with the DPPH assay remained stable over time (for several days).

Applying the ABTS assay, the antioxidant capacity showed higher values than the DPPH assay, with values that ranged between 382.4 (1st day) and 615.4 mmol TE kg^−1^ FW (7 days, in E sample). The TAA tended to increase during the storage of the fruit, particularly in samples A, B, C, and E, while the other three samples did not show significant differences during the time period (*p* > 0.05).

## 4. Conclusions

The application of enriched gum Arabic coating could be a valid alternative during fruit storage, providing beneficial effects by retarding the ripening process. The results obtained in this experimentation process confirmed the positive actions of the coatings, creating good conditions such as an increase in storage time as compared with the control sample and the samples coated with a synthetic antioxidant. In fact, the control sample deteriorated rapidly. The application of edible coatings provided a useful barrier to preserve the antioxidant parameters, delaying ripening and senescence. The enriched coatings can retain the quality parameters in strawberries after prolonged refrigerated storage.

After 14 days of storage, the samples that showed the best qualitative characteristics were those coated with the antioxidant extract at 2.5% (D) and with bergamot essential oil at 0.1% (F). For these samples, lower decay rates were observed (36% for sample D and 27% for sample F), with better acceptability from sensory and textural points of view, with scores above 4.5, which represents the limit of acceptability (appearance scores of 6, turgidity scores of 6 and 5, and overall acceptability scores of 6 and 5, respectively, for samples D and F), as well as showing good maintenance of the organic acids, especially ascorbic acid (31.47 for sample D and 30.80 mg 100 g^−1^ for sample F), an indicator of quality. This experimentation process revealed good results compared to the normal shelf life of strawberries.

The use of a natural antioxidant extract and bergamot essential oil in the coating formulation revealed the good possibility to achieve the double effect of barrier resistance to respiration and transpiration and an antimicrobial effect against microbial growth during shelf life, preserving fruit quality.

## Figures and Tables

**Table 1:** Sample denomination.

Sample	Enriching Antioxidant Compounds
A	none
B	BHT (100 ppm)
C	AE (1%)
D	AE (2.5%)
E	AE (5%)
F	BEO (0.1% + Tween 20)
G	BEO (0.2% + Tween 20)

**Table 2:** Physicochemical characterization of antioxidant extract from bergamot pomace (AE).

pH	3.18 ± 0.08
TSS (°Brix)	21.1 ± 0.85
L*	49.33 ± 0.22
a*	0.57 ± 0.09
b*	2.83 ± 0.14
TPC (mg GAE L^−1^)	7751 ± 137
TFC (mg CE L^−1^)	2783 ± 30
DPPH (mmol TE L^−1^)	359 ± 2
ABTS (mmol TE L^−1^)	2100 ± 16
p-cumaric acid (mg L^−1^)	116.77 ± 5.58
Ferulic acid (mg L^−1^)	38.02 ± 1.69
Eriocitrin (mg L^−1^)	75.18 ± 5.15
Neoeriocitrin (mg L^−1^)	4194.78 ± 59.68
Narirutin (mg L^−1^)	29.72 ± 5.08
Naringin (mg L^−1^)	3544.51 ± 114.73
Neohesperidin (mg L^−1^)	2219.09 ± 4.32
Melitidin (mg L^−1^)	821.54 ± 70.66
Brutieridin (mg L^−1^)	1782.75 ± 12.74

**Table 3:** Chemical composition of bergamot essential oil (BEO).

Total Antioxidants Properties		
DPPH (mmol TE L^−1^)	267.04 ± 21.21	
ABTS (mmol TE L^−1^)	230.82 ± 18.61	
**Volatile fraction**	**rt**	**%**
1 Tricyclene	5.65	0.001
2 α-Thujene	5.77	0.210
3 α-Pinene	6.04	0.839
4 Camphene	6.59	0.016
5 Sabinene	7.62	0.590
6 β-Pinene	7.81	3.322
7 Myrcene	8.44	1.058
8 Octanal	9.10	0.023
9 α-Phellandrene	9.20	0.024
10 δ-3-Carene	9.45	0.001
11 α-Terpinene	9.96	0.103
12 p-Cymene	10.51	0.025
13 Limonene	11.04	46.727
14 (Z)-β-Ocimene	11.39	0.013
15 (E)-β-Ocimene	12.02	0.136
16 γ-Terpinene	12.74	5.432
17 trans-Sabinene hydrate	13.20	0.025
18 Octanol	13.63	0.002
19 Terpinolene	14.48	0.235
20 Linalool	15.42	4.386
21 Nonanal	15.57	0.016
22 cis-Limonene oxide	16.17	0.003
23 trans-Limonene oxide	16.26	0.003
24 Isopulegol	17.49	0.002
25 Camphor	17.80	0.002
26 Citronellal	18.43	0.010
27 Terpinen-4-ol	19.72	0.014
28 α-Terpineol	20.49	0.035
29 Decanal	21.40	0.031
30 Octyl acetate	21.81	0.072
31 Nerol	22.63	0.042
32 Neral	23.27	0.205
33 cis-Sabinene hydrate acetate	23.92	0.051
34 Linalyl acetate	24.33	34.151
35 Geranial	24.84	0.312
36 α-Terpinyl acetate	28.62	0.119
37 Citronellyl acetate	28.87	0.029
38 Neryl acetate	29.38	0.502
39 Geranyl acetate	30.27	0.232
40 Dodecanal	31.43	0.027
41 Decyl acetate	31.57	0.013
42 (β)-Caryophyllene	31.71	0.215
43 trans-α-Bergamotene	32.42	0.247
44 α-Humulene	33.11	0.015
45 cis-β-Farnesene	33.28	0.045
46 Germacrene D	34.19	0.039
47 Sesquiterpene	34.34	0.012
48 Sesquiterpene	34.78	0.016
49 α-Farnesene	35.02	0.024
50 β-Bisabolene	35.24	0.349

**Table 4:** Weight loss (%) values of the strawberries during storage days.

Sample/Time	1	3	7	10	14	Sign.
A	0.55 ± 0.06 ^aCE^	1.51 ± 0.11 ^aD^	3.12 ± 0.14 ^aC^	9.27 ± 0.08 ^aB^	25.38 ± 2.45 ^aA^	**
B	0.52 ± 0.03 ^abD^	1.02 ± 0.03 ^bC^	1.26 ± 0.03 ^dC^	8.33 ± 0.10 ^cB^	22.09 ± 0.39 ^abA^	**
C	0.42 ± 0.03 ^cE^	0.95 ± 0.02 ^cDE^	2.11 ± 0.08 ^cC^	7.49 ± 0.06 ^dB^	19.77 ± 4.48 ^abA^	**
D	0.31 ± 0.04 ^dD^	0.98 ± 0.03 ^bcC^	0.99 ± 0.04 ^eC^	4.19 ± 0.08 ^gB^	12.28 ± 3.86 ^bA^	**
E	0.38 ± 0.04 ^cE^	0.93 ± 0.03 ^cdD^	1.2 ± 0.05 ^dC^	6.34 ± 0.07 ^eB^	16.41 ± 1.54 ^bA^	**
F	0.29 ± 0.03 ^dD^	0.86 ± 0.01 ^dC^	0.91 ± 0.04 ^eC^	5.31 ± 0.08 ^fB^	12.78 ± 2.93 ^bA^	**
G	0.48 ± 0.02 ^bE^	0.97 ± 0.02 ^bcD^	2.37 ± 0.10 ^bC^	8.78 ± 0.14 ^bB^	20.46 ± 2.76 ^abA^	**
Sign.	**	**	**	**	**	

Small letters within a row and capital letters within a column show significant differences as assessed by Tukey’s post hoc test. Abbreviations: **, significance at *p* < 0.01.

**Table 5:** Surface color values of minimally treated strawberries.

	A	B	C	It D	E	F	G	Sign.
				L*				
1st	49.9 ± 2.3 ^aA^	47.2 ± 1.4 ^B^	47.3 ± 1.5 ^B^	46.7 ± 2.2 ^B^	47.4 ± 1.4 ^B^	46.7 ± 1.2 ^B^	47.4 ± 1.5 ^B^	**
7th	46.4 ± 1.4 ^b^	47.3 ± 2.6	46.6 ± 1.0	45.8 ± 2.1	46.3 ± 0.8	47.4 ± 2.4	47.0 ± 2.2	n.s.
14th	46.6 ± 1.0 ^b^	47.8 ± 1.4	47.9 ± 1.6	48.6 ± 4.4	47.2 ± 1.3	47.3 ± 1.2	47.8 ± 1.8	n.s.
Sign.	**	n.s.	n.s.	n.s.	n.s.	n.s.	n.s.	
a*								
1st	17.6 ± 3.0 ^aA^	13.0 ± 2.2 ^B^	15.8 ± 2.8 ^AB^	13.0 ± 3.8 ^B^	14.6 ± 3.0 ^AB^	13.0 ± 2.3 ^B^	13.8 ± 3.1 ^AB^	**
7th	13.9 ± 3.6 ^b^	15.8 ± 3.8	15.0 ± 2.1	15.4 ± 4.6	15.4 ± 2.3	16.2 ± 3.7	16.1 ± 4.2	n.s.
14th	11.3 ± 2.6 ^b^	14.3 ± 3.8	14.2 ± 3.4	14.6 ± 4.2	14.1 ± 3.2	15.8 ± 3.0	14.9 ± 2.0	n.s.
Sign.	**	n.s.	n.s.	n.s.	n.s.	n.s.	n.s.	
b*								
1st	10.5 ± 3.0 ^aA^	7.3 ± 2.1 ^B^	8.4 ± 2.3 ^AB^	7.55 ± 2.3 ^AB^	8.1 ± 2.2 ^AB^	6.6 ± 1.4 ^B^	7.0 ± 2.1 ^B^	**
7th	7.0 ± 2.1b	8.1 ± 3.9	7.3 ± 1.0	7.4 ± 2.5	7.1 ± 1.1	7.9 ± 2.6	8.4 ± 2.9	n.s.
14th	4.8 ± 1.4c	6.6 ± 2.4	7.0 ± 2.7	7.7 ± 3.8	6.4 ± 1.8	7.3 ± 1.7	6.9 ± 2.0	n.s.
Sign.	**	n.s.	n.s.	n.s.	n.s.	n.s.	n.s.	
C*								
1st	20.5 ± 4.1 ^aA^	14.9 ± 2.8 ^B^	17.9 ± 3.5 ^AB^	15.1 ± 4.3 ^B^	16.7 ± 3.7 ^AB^	14.6 ± 2.5 ^B^	15.5 ± 2.7 ^B^	**
7th	15.6 ± 4.0 ^b^	17.9 ± 5.2	16.7 ± 2.3	17.1 ± 5.1	16.9 ± 2.6	18.1 ± 4.4	18.2 ± 5.0	n.s.
14th	12.2 ± 2.9 ^c^	15.7 ± 4.4	15.8 ± 4.2	16.6 ± 5.3	15.5 ± 3.7	17.4 ± 3.4	16.4 ± 2.6	n.s.
Sign.	**	n.s.	n.s.	n.s.	n.s.	n.s.	n.s.	
h°								
1st	30.3 ± 3.6 ^a^	28.9 ± 3.9 ^a^	27.8 ± 3.2	30.1 ± 3.6	28.6 ± 2.4 ^a^	26.9 ± 3.0 ^a^	26.7 ± 1.7	n.s.
7th	26.7 ± 2.7 ^b^	26.3 ± 4.5 ^ab^	26.1 ± 1.8	25.8 ± 3.5	24.7 ± 1 ^b^	25.4 ± 2.5 ^ab^	27.3 ± 3.3	n.s.
14th	22.7 ± 1.7 ^c^	24.3 ± 2.3 ^b^	25.5 ± 3.5	26.9 ± 5.6	24.3 ± 1.2 ^b^	24.6 ± 2.1 ^b^	24.7 ± 3.5	n.s.
Sign.	**	*	n.s.	n.s.	**	*	n.s.	

Small letters within a column and capital letters within a row show significant differences as assessed by Tukey’s post hoc test. Abbreviations: **, significance at *p* < 0.01; *, significance at *p* < 0.05; n.s., not significant.

**Table 6:** Sensorial characterization of minimally treated strawberries.

	Appearance		Aroma Intensity		Sweetness		Turgidity		Overall Acceptability	
	1st	14th	1st	14th	1st	14th	1st	14th	1st	14th
A	8.5 ^a^	4 ^b^	6.5 ^a^	4 ^ab^	4 ^b^	3 ^c^	7 ^a^	3 ^c^	6 ^b^	3 ^d^
B	7 ^b^	4 ^b^	3 ^c^	1 ^c^	6 ^a^	3 ^c^	6 ^b^	5 ^ab^	7 ^a^	3 ^d^
C	8 ^ab^	4.5 ^b^	4 ^b^	3 ^b^	5 ^ab^	4 ^b^	6 ^b^	4 ^b^	7 ^a^	4 ^c^
D	8 ^ab^	6 ^a^	5 ^ab^	3 ^b^	5 ^ab^	3 ^c^	7 ^a^	6 ^a^	7 ^a^	6 ^a^
E	7 ^b^	6 ^a^	4 ^b^	3 ^b^	5.5 ^a^	5 ^a^	7 ^a^	5 ^ab^	7 ^a^	5.5 ^ab^
F	7 ^b^	6 ^a^	3 ^c^	4 ^ab^	5 ^ab^	3 ^c^	6.5 ^ab^	5 ^ab^	6.5 ^ab^	5 ^b^
G	7 ^b^	6 ^a^	5 ^ab^	5 ^a^	4.5 ^b^	3 ^c^	6.5 ^ab^	5.5 ^a^	7 ^a^	4 ^c^
Sign.	*	**	**	**	**	**	*	**	*	**

Small letters within a column show significant differences as assessed by Tukey’s post hoc test. Abbreviations: **, significance at *p* < 0.01; *, significance at *p* < 0.05.

**Table 7:** The pH, TA (% citric acid), and organic acid contents (ascorbic acid, AA; mg 100 g^−1^; citric acid, CA; mg 100 g^−1^) of strawberries.

pH	A	B	C	D	E	F	G	Sign.
1st	3.4 ± 0 ^b^	3.4 ± 0.0 ^c^	3.4 ± 0.0 ^b^	3.4 ± 0.0 ^b^	3.4 ± 0.0 ^b^	3.4 ± 0.0 ^c^	3.4 ± 0.0 ^c^	ns
7th	4.0 ± 0.1 ^aA^	3.7 ± 0.0 ^bB^	3.6 ± 0.0 ^aC^	3.6 ± 0.2 ^aC^	3.6 ± 0.0 ^aC^	3.7 ± 0.0 ^bC^	3.6 ± 0.0 ^bC^	**
14th	4.0 ± 0 ^aA^	4.0 ± 0.0 ^aA^	3.8 ± 0.0 ^aC^	3.6 ± 0.0 ^aD^	3.7 ± 0.0 ^aCD^	3.9 ± 0.0 ^aB^	3.7 ± 0.0 ^aC^	**
Sign.	**	**	**	**	**	**	**	
TA	A	B	C	D	E	F	G	Sign.
1st	0.7 ± 0.0 ^b^	0.7 ± 0.0	0.7 ± 0.0 ^b^	0.7 ± 0.0	0.7 ± 0.0	0.7 ± 0.0 ^b^	0.7 ± 0.0 ^b^	ns
7th	0.8 ± 0.0 ^aA^	0.7 ± 0.1 ^B^	0.9 ± 0.1 ^aA^	0.7 ± 0.1 ^B^	0.7 ± 0.1 ^B^	0.8 ± 0.1 ^abAB^	0.9 ± 0.1 ^aA^	*
14th	0.5 ± 0.0 ^cD^	0.7 ± 0.0 ^C^	0.9 ± 0.0 ^aA^	0.9 ± 0.1 ^A^	0.8 ± 0.0 ^B^	0.8 ± 0.1 ^aB^	0.9 ± 0.0 ^aAB^	*
Sign.	**	n.s.	**	n.s.	n.s	*	**	
AA	A	B	C	D	E	F	G	Sign.
1st	33.0 ± 0.5 ^a^	33.0 ± 0.5 ^a^	33.0 ± 0.5 ^a^	33.0 ± 0.5 ^a^	33.0 ± 0.5 ^a^	33.0 ± 0.5 ^a^	33.0 ± 0.5 ^a^	ns
7th	28.4 ± 0.1 ^bCD^	27.3 ± 0.4 ^cD^	32.6 ± 0.1 ^aA^	29.6 ± 0.8 ^bBC^	31.4 ± 1.02 ^abAB^	28.5 ± 0.2 ^bCD^	30.1 ± 0.2 ^bBC^	**
14th	27.0 ± 0.2 ^cB^	29.1 ± 0.3 ^bAB^	30.3 ± 0.1 ^bA^	31.5 ± 1.4 ^abA^	29.8 ± 0.5 ^bAB^	30.8 ± 1.4 ^abA^	29.9 ± 0.4 ^bAB^	*
Sign.	**	**	**	*	*	**	**	
CA	A	B	C	D	E	F	G	Sign.
1st	692.5 ± 26.5 ^a^	692.5 ± 26.5 ^a^	692.5 ± 26.5	692.5 ± 26.5	692.5 ± 26.5	692.5 ± 26.5	692.50 ± 26.5 ^a^	ns
7th	604.1 ± 42.0 ^aC^	669.1 ± 2.2 ^aABC^	720.5 ± 10.2 ^A^	745.7 ± 2.1 ^A^	698.0 ± 12.3 ^AB^	699.5 ± 30.8 ^AB^	636.2 ± 41.6 ^abBC^	*
14th	402.9 ± 3.3 ^bB^	583.5 ± 56.0 ^bA^	711.1 ± 20.3 ^A^	727.5 ± 32.1 ^A^	676.8 ± 17.9 ^A^	654.5 ± 85.42 ^A^	558.6 ± 43.3 ^bAB^	*
Sign.	**	**	ns	ns	ns	ns	*	

Small letters within a column (among storage time) and capital letters within a row (among different treated samples) show significant differences as assessed by Tukey’s post hoc test. Abbreviations: **, significance at *p* < 0.01; *, significance at *p* < 0.05; n.s., not significant.

**Table 8:** The microbiological counts of minimally treated strawberries (Log10 CFU g^−1^).

	CBT				Yeasts				Molds			
	1st	7th	14th	Sign.	1st	7th	14th	Sign.	1st	7th	14th	Sign.
A	1.8 ^aC^	2.9 ^aB^	3.4 ^bA^	**	1.1 ^aC^	4.9 ^bB^	7.0 ^aA^	**	3.0 ^aB^	3.5 ^aB^	5.3 ^aA^	**
B	1.0 ^bC^	2.0 ^bB^	4.1 ^aA^	**	1.1 ^aB^	6.1 ^aA^	6.4 ^bA^	**	2.1 ^bB^	2.1 ^bB^	5.3 ^aA^	**
C	0 ^cC^	2.5 ^abB^	3.7 ^abA^	**	0 ^bB^	5.8 ^aA^	5.2 ^cA^	**	0 ^cB^	0 ^cB^	4.4 ^bA^	**
D	0 ^cB^	2.9 ^aA^	2.4 ^cA^	**	0 ^bB^	4.9 ^bA^	6.4 ^bA^	**	0 ^cC^	2.1 ^bB^	4.8 ^abA^	**
E	0 ^cC^	1.3 ^cB^	3.1 ^bA^	**	0 ^bC^	1.5 ^dB^	4.7 ^cA^	**	0 ^cB^	0 ^cB^	3.2 ^cA^	**
F	0 ^cB^	2.3 ^abA^	2.0 ^cdA^	**	0 ^bB^	2.6 ^cA^	2.3 ^dA^	**	0 ^cB^	2.2 ^bA^	1.9 ^dA^	**
G	0 ^cB^	1.7 ^bcA^	1.8 ^dA^	**	0 ^bB^	2.7 ^cA^	2.3 ^dA^	**	0 ^cB^	0 ^cB^	1.5 ^dA^	**
Sign.	**	**	**		**	**	**		**	**	**	

Small letters within a column and capital letters within a row show significant differences as assessed by Tukey’s post hoc test. Abbreviations: **, significance at *p* < 0.01.

**Table 9:** Total antioxidant compound levels of minimally treated strawberries.

	TPC (mg GAE 100 g^−1^ FW)							
	A	B	C	D	E	F	G	Sig
1st	101.2 ± 2.8	101.2 ± 2.8 ^b^	101.2 ± 2.8 ^b^	101.2 ± 2.8 ^b^	101.2 ± 2.8 ^b^	101.2 ± 2.8 ^b^	101.2 ± 2.8 ^b^	ns
7th	106.8 ± 5.7	118.1 ± 4.1 ^a^	118.7 ± 5.7 ^a^	122.6 ± 4.8 ^a^	125.0 ± 8.4 ^a^	119.7 ± 1.7 ^a^	127.0 ± 16.0 ^a^	ns
14th	108.2 ± 4.0	115.8 ± 3.8 ^a^	119.5 ± 6.1 ^a^	115.6 ± 17.0 ^ab^	115.0 ± 16.3 ^ab^	117.8 ± 3.4 ^a^	109.7 ± 1.7 ^ab^	ns
Sig.	ns	**	**	*	*	**	*	
	TFC (mg CE 100 g^−1^ FW)							
1st	20.2 ± 1.1 ^b^	20.2 ± 1.1 ^b^	20.2 ± 1.1 ^b^	20.2 ± 1.1 ^b^	20.2 ± 1.1 ^b^	20.2 ± 1.1 ^b^	20.2 ± 1.1 ^b^	ns
7th	19.9 ± 1.1 ^bB^	18.7 ± 0.5 ^bB^	21.2 ± 1.3 ^bB^	19.4 ± 3.4 ^bB^	22.0 ± 0.7 ^bB^	20.3 ± 1.0 ^B^	26.5 ± 1.1 ^aA^	**
14th	32.9 ± 1.6 ^aABC^	35.8 ± 2.1 ^aA^	34.2 ± 1.1 ^aAB^	32.6 ± 5.8 ^aABC^	27.6 ± 3.9 ^aCD^	23.7 ± 4.8 ^D^	26.2 ± 0.8 ^aCD^	**
Sig.	**	**	**	**	**	n.s.	**	
	TAC (mg PGN 100 g^−1^ FW)							
1st	20.34 ± 0.43 ^b^	20.34 ± 0.43 ^b^	20.34 ± 0.43	20.34 ± 0.43 ^b^	20.34 ± 0.43 ^b^	20.34 ± 0.43 ^c^	20.34 ± 0.43	ns
7th	33.74 ± 1.36 ^aA^	24.70 ± 0.73 ^aC^	21.84 ± 0.76 ^D^	24.75 ± 1.06 ^aBC^	24.20 ± 1.17 ^aC^	26.90 ± 0.87 ^aB^	18.09 ± 0.24 ^E^	**
14th	17.52 ± 3.68 ^bC^	21.16 ± 2.55 ^bABC^	22.74 ± 2.68 ^ABC^	23.94 ± 1.04 ^aAB^	24.96 ± 2.95 ^aA^	24.85 ± 0.93 ^bA^	18.44 ± 2.79 ^BC^	**
Sig.	**	**	ns	**	*	**	n.s.	
	DPPH (mmol TE kg^−1^ FW)							
1st	145.9 ± 9.8	145.9 ± 9.8 ^ab^	145.9 ± 9.8 ^b^	145.9 ± 9.8	145.9 ± 9.8 ^b^	145.9 ± 9.8 ^b^	145.9 ± 9.8	ns
7th	156.8 ± 15.1 ^BC^	129.9 ± 5.0 ^bC^	189.4 ± 19.8 ^bAB^	164.6 ± 13.6 ^BC^	205.1 ± 14.4 ^aA^	215.5 ± 12.1 ^aA^	153.5 ± 19.3 ^BC^	**
14th	161.1 ± 8.5	160.1 ± 13.5 ^a^	149.9 ± 20.3 ^a^	145.9 ± 19.2	131.6 ± 10.7 ^b^	153.7 ± 5.9 ^b^	153.7 ± 7.1	ns
Sig.	n.s.	**	*	n.s.	**	**	n.s.	
	ABTS (mmol TE kg^−1^ FW)							
1st	382.4 ± 7.8 ^b^	382.4 ± 7.8 ^b^	382.4 ± 7.8 ^b^	382.4 ± 7.8	382.4 ± 7.8 ^b^	382.4 ± 7.8	382.4 ± 7.8	ns
7th	436.9 ± 36.4 ^aBC^	504.1 ± 37.0 ^aB^	448.8 ± 31.7 ^abBC^	364.5 ± 54.9 ^C^	615.4 ± 42.5 ^aA^	386.2 ± 35.3 ^C^	438.5 ± 44.8 ^BC^	**
14th	449.6 ± 14.8 ^aAB^	511.7 ± 51.5 ^aA^	511.3 ± 73.5 ^aA^	412.8 ± 58.9 ^AB^	425.7 ± 39.2 ^bAB^	391.3 ± 18.4 ^B^	419.1 ± 36.4 ^AB^	**
Sig.	**	**	*	n.s.	**	n.s.	n.s.	

Small letters within a column and capital letters within a row show significant differences as assessed by Tukey’s post hoc test. Abbreviations: **, significance at *p* < 0.01; *, significance at *p* < 0.05; n.s., not significant.

## Data Availability

The datasets generated for this study are available on request to the corresponding author.
